# Supplementary material for: Genetic Structure and Diversity of Hatchery and Wild Populations of Yellow Catfish Tachysurus fulvidraco (Siluriformes: Bagridae) from Korea
Source: Int J Mol Sci. 2024 Mar 31;25(7):3923. doi: 10.3390/ijms25073923 (PMC11011370; doi:10.3390/ijms25073923)
Supplement: Supplementary file 1 [file ijms-25-03923-s001.zip › ijms-2912948-supplementary.pdf]

Table S1. Summary information of ms DNA screening and raw data of sequencing of *Tachysurus fulvidraco.*

	Pfu01	Pfu02	Pfu03	Pfu04
Raw data number of read	6,231,011	5,705,048	5,123,581	6,235,796
Raw data number of bases (bp)	3,751,068,622	3,434,438,896	3,084,395,762	3,753,949,192
After trimming sequences (bp)	3,460,047,409	3,119,844,627	2,670,567,764	3,487,610,285
Total number of sequences examined	12,356,363	11,332,513	10,136,401	12,375,977
Total size of examined sequences (bp)	3,450,011,112	3,111,973,483	2,662,086,408	3,479,909,293
Total number of identified SSRs	2,805,105	3,218,502	4,232,443	2,892,730
Number of SSR containing franking sequences	1,803,312	1,979,878	2,412,325	1,841,306
Number of sequences containing more than 1 SSR	603,858	729,548	1,030,803	628,504
Di-nucleotide	2,065,925	2,515,694	3,560,225	2,065,989
Tri-nucleotide	275,480	253,883	230,434	319,490
Tetra-nucleotide	415,295	399,537	392,783	454,810
Penta-nucleotide	31,639	32,161	30,143	37,837
Hexa-nucleotide	16,766	17,227	18,858	14,604

Table S2. Summary information of the 15 ms DNA markers developed of *Tachysurus fulvidraco*.

Primer name	Length (mer)	SSR Type	Amplicon Size (bp)
Pfu-P2-002	22	$(GT)_{10}$	152
Pfu-P2-003	22	$(TG)_{19}$	187
Pfu-P2-005	22	$(TG)_{15}$	202
Pfu-P2-006	20	$(TG)_{14}$	205
Pfu-P2-009	18	$(TC)_{15}$	241
Pfu-P2-011	19	$(TC)_{14}$	253
Pfu-P2-015	21	$(TG)_{20}$	279
Pfu-P2-016	21	$(AC)_{16}$	280
Pfu-P2-018	20	$(TC)_{20}$	293
Pfu-P3-003	18	$(AGA)_{11}$	182
Pfu-P3-004	18	$(AGA)_{20}$	185
Pfu-P3-008	25	$(AAG)_{16}$	243
Pfu-P4-001	22	$(TAAA)_{10}$	128
Pfu-P4-002	20	$(TAAA)_{9}$	128
Pfu-P4-005	18	$(ATAC)_{7}$	197

Table S3. Summary of msDNA multiplex PCR set in *Tachysurus fulvidraco.*

Microsatellite multiplex PCR set 1

Dye	Name	Forward sequence	Reverse sequence
FAM	Pfu-P2-002	ACACGTAACTGTTTATCTGTGC	ACAGCCATGAGAGGAAGC
	Pfu-P2-015	TGAAATCTATCAGTGGCTCCA	CTAACTGCCATCTAACTGAGG
HEX	Pfu-P2-006	ACACTGGTTTGTTGTGTAGC	ACGCTGAACAATGGACA
	Pfu-P2-003	ACCACACTTCTAAAAAGAAAATGTT	GCAAAAAATGAGCTGGCTGA
TAMRA	Pfu-P2-009	ACGACAGCGATAGGAGGT	GGGAGGAAGGGAGTGATT
	Pfu-P2-018	TACACAAACATGTCGAACGG	AGATGCACTTGAAGGAACCT
ATTO565	Pfu-P3-003	ACCTCGTTTTCCTGCTCA	GCAGCCCTCCTGTTCTTC
	Pfu-P2-016	TTGTTTCACAGATGTTTCGTG	GACGACACAGGACTACTG

Microsatellite multiplex PCR set 2

Dye	Name	Forward sequence	Reverse sequence
FAM	Pfu-P3-004	AACGCACTACCTGTTCCT	GCCAATGCTGGATTTGTC
	Pfu-P4-002	ATCTGATTACGGGTCGAAAC	GAGAGCCGCTACAACACT
HEX	Pfu-P3-008	TGGTTTGCTGCTGTGCT	ATTCCTGCCTGCATCTCA
	Pfu-P2-011	AGAACACACAGGATTCAGC	GCTAGTGTTAGTATCTTCCATCT
TAMRA	Pfu-P4-005	TTGTCGTACGGACTGCTT	AAGGCTTTTCCACCTGCA
	Pfu-P2-005	TGTTCTGTCAAAGTAGAAGGTT	AGCCATAGTGATCCTGAAC
ATTO565	Pfu-P4-001	CATTACACGCCTGTTTGTTTAC	AGCGATGAATGATATCTTTGAAA

Table S4. Polymorphism of genetic diversity information of msDNA loci in *Tachysurus fulvidraco*.

Locus	N	N_A	H_O	H_E	P_{IC}	Null allele
Pfu-P2-002	120	13	0.742	0.797	0.769	X
Pfu-P2-015	120	18	1.000	0.865	0.848	X
Pfu-P2-006	120	11	0.783	0.852	0.834	X
Pfu-P2-003	120	11	0.308	0.617	0.563	X
Pfu-P2-009	120	15	1.000	0.889	0.875	X
Pfu-P2-018	120	15	0.875	0.892	0.878	X
Pfu-P3-003	120	12	0.925	0.844	0.823	X
Pfu-P2-016	120	18	0.950	0.867	0.850	X
Pfu-P3-004	120	13	0.833	0.845	0.825	X
Pfu-P4-002	120	11	0.733	0.850	0.828	X
Pfu-P3-008	120	19	0.800	0.897	0.884	X
Pfu-P2-011	120	11	0.800	0.832	0.809	X
Pfu-P4-005	120	13	0.992	0.840	0.820	X
Pfu-P2-005	120	9	0.758	0.795	0.766	X
Pfu-P4-001	120	12	0.783	0.830	0.807	X

N: Number of samples, N_A: Number of alleles, H_O: Observed heterozygosity, H_E: Expected heterozygosity, P_{IC}: Polymorphic information content.
